# Complete blood count reference intervals for extremely preterm neonates

**DOI:** 10.1007/s00431-025-06544-4

**Published:** 2025-10-18

**Authors:** Christina H. Wolfsberger, Benedikt N. Seidel, Linda Fleck, Markus Herrmann, Diether Kramer, Martin Benesch, Gerhard Pichler, Markus G. Seidel

**Affiliations:** 1 Department of Pediatrics and Adolescent Medicine, Division of Neonatology, Medical University of Graz, Graz, Austria; 2 Styrian Children’s Cancer Research Unit for Cancer and Inborn Errors of the Blood and Immunity in Children, Medical University of Graz, Graz, Austria; 3 Autonomous Aerial Systems, School of Engineering and Design, Technical University of Munich, Munich, Germany; 4 Clinical Institute of Medical and Chemical Laboratory Diagnostics, Medical University of Graz, Graz, Austria; 5 Directorate for Technology and IT, IT Management Team, Styrian Hospital Association (KAGes), Graz, Austria; 6 Department of Pediatrics and Adolescent Medicine, Division of Pediatric Hematology-Oncology, Medical University of Graz, Graz, Austria

**Keywords:** Preterm infant, Newborn infant, Reference intervals, Centile charts, Full blood counts (FBC), Complete blood counts (CBC), Normal range, Lymphocytes

## Abstract

Preterm infants exhibit dynamic hematological profiles during the first postnatal days, necessitating robust reference intervals for clinical interpretation. The aim of this study is to establish comprehensive reference intervals for complete blood counts in preterm neonates during the first five postnatal days, stratified by gestational age and postnatal age. Data from preterm neonates (23 + 0–34 + 0 weeks gestation) admitted to the neonatal intensive care unit Graz (2008–2023) were analyzed. Percentiles (2.5th, 10th, 50th, 90th, 97.5th) were generated for the following hematological parameters included in the full/complete and differential blood count (FBC, CBC): leukocytes, thrombocytes, hemoglobin, hematocrit, neutrophilic granulocytes, and lymphocytes. Stringent exclusion criteria minimized confounding factors. Comparisons were made between appropriate-for-gestational age (AGA) and small-for-gestational age (SGA) neonates. A total of 3128 eligible preterm neonates with a median (minimum; maximum) gestational age of 32.1 (23.1; 34.0) weeks and a birth weight of 1700 (297; 3210) grams were included for analysis. Reference intervals were established for each parameter across gestational ages and postnatal days. Significant differences were observed between AGA and SGA neonates, particularly for leukocytes, thrombocytes, and neutrophilic granulocyte with lower values in SGA infants. Hemoglobin concentration and hematocrit were largely similar between groups, with some exceptions at specific gestational ages.

*Conclusion*: This study provides reference intervals for hematological parameters in preterm neonates, enabling improved clinical interpretation and identification of deviations. The large sample size and strict exclusion criteria enhance the validity of these reference intervals. The observed differences between AGA and SGA neonates warrant further investigation to understand the clinical implications of fetal growth restriction on neonatal hemato-/lymphopoiesis.

**Supplementary Information:**

The online version contains supplementary material available at 10.1007/s00431-025-06544-4.

**Table:** 

**What is Known:** • *Existing neonatal hematologic reference intervals often lack stratification by gestational age and fetal growth*. • *This limits their utility in preterm and small-for-gestational-age infants, who have unique hematologic profiles due to distinct developmental trajectories and potential perinatal complications*. **What is New:** • *This study establishes hematologic reference intervals stratified by gestational age and postnatal age specifically for preterm neonates*. •* It further highlights key differences between appropriate-for-gestational age and small-for-gestational age infants, improving the diagnostic accuracy for this vulnerable population*.	

## Introduction

Prematurity, defined as birth before 37 weeks of gestational age, is associated with various physiological and pathophysiological differences compared to term born neonates. Besides morbidities such as bronchopulmonary dysplasia, retinopathy of prematurity, and intraventricular hemorrhage [1], preterm infants also exhibit variations in blood counts and immune function compared to term neonates [2].

Hematopoiesis in preterm neonates differs significantly due to the ongoing maturation of the hematopoietic system after birth. During (embryo- and) fetogenesis, hematopoiesis primarily occurs in the liver and spleen, gradually shifting to the bone marrow as the main site of blood cell production [3]. This transition is often incomplete in preterm neonates, leading to lower blood cell counts. Red blood cell, leukocyte, and thrombocyte production is influenced by gestational age, postnatal age, and medical conditions. Understanding these characteristics is crucial for clinical management and optimizing outcomes [3, 4].

Hematopoiesis and immune development continue postnatally in preterm neonates leading to dynamic changes during the first weeks [2]. Hemoglobin levels decline after birth, due to a physiological transient decrease in erythropoietin, especially in preterm neonates, resulting in reduced regenerative capacity [4]. Platelet counts can be low (thrombocytopenia), though clinically significant thrombocytopenia is less common. Gestational and postnatal age, along with comorbidities, influence platelet counts [5, 6]. Leukocyte counts fluctuate, initially rising then declining [7]. Lymphocyte counts are generally lower in preterm neonates, reflecting a immune immaturity [8], with both number and function of T lymphocytes impaired. This contributes to a higher susceptibility of preterm neonates to infections and underscores the need for protective measures in their care.

Although changes in blood count values in preterm neonates have been reported [9], detailed information on specific components—leukocytes, thrombocytes, hemoglobin, hematocrit, neutrophilic granulocytes, and lymphocytes—in relation to gestational/postnatal age, and intrauterine growth restriction (IUGR) or small-for-gestational age (SGA) status, are still lacking.

This study aimed to define reference intervals for blood cell counts in neonates born before 34 weeks of gestation during the first 5 days after birth, stratified by gestational and postnatal age. We hypothesized that normative values would vary with gestational/postnatal age and differ between neonates born appropriate-for-gestational age (AGA) and SGA/IUGR neonates.

## Methods

### Study design

The study was designed as a retrospective single-center study, conducted at the Department of Pediatrics and Adolescent Medicine, Medical University of Graz. Data of preterm neonates born between January 2008 and December 2023 were eligible for analysis. The study was approved by Regional Committee on Biomedical Research Ethic of the Medical University of Graz (EC number: 36–215 ex 23/24) and was carried out in accordance with The Code of Ethics of the World Medical Association (Declaration of Helsinki) [10].

### Population

Preterm neonates with a gestational age of 34 + 0 weeks or less admitted to the neonatal intensive care unit in Graz were included in this study. Blood samples collected within the first 7 days after birth were analyzed. Exclusion factors are presented in Online Supplement Table 1.

### Data analysis

The antepartum medical history (maternal peripartal infection, preterm premature rupture of membranes, IUGR, amniotic fluid infection, gestosis]), antenatal steroids, fetal infections (fetal cytomegalovirus, fetal toxoplasmosis infection, fetal parvovirus B19 infection), fetal proven inborn error of immunity, fetal genetic abnormality, positive family history of severe combined immunodeficiency (SCID), and demographic data (gestational age, gender birth weight, length, diagnosis of SGA [defined as birth weight < 10th centile] or IUGR, Apgar score, umbilical cord pH, mode of delivery and causes of preterm birth) were documented. Received medication during the first 7 days after birth at the NICU, including antibiotics, immunoglobulins, transfusion of platelets/red blood cells, G-CSF, anticonvulsants, steroids, non-steroidal antirheumatic drugs including patent ductus arteriosus-related treatments were documented. Short-term outcome parameters, defined as any diagnosis at 37 to 42 weeks of gestational age or time point of discharge, were assessed. Further, any diagnosis of SCID, infection with opportunistic germs, or of a hematological disease including leukemia/lymphoma or autoimmunocytopenia until an age of corrected 5 years were noted.

Routinely performed blood samples of the preterm neonate were documented within the first 5 days after birth, focusing on leukocytes, thrombocytes, hemoglobin, hematocrit, absolute number of neutrophilic granulocytes, and absolute number of lymphocytes. The documentation included the method of blood sampling, and specifying whether it was an arterial, venous, or capillary sample. All blood sample were measured with a Sysmex XN (Sysmex Austria, Vienna) analyser in our ISO 15189 accredited central laboratory. Intra- and interassay imprecision for all analyses and levels were < 4.7% (1.2% to 4.7%).

### Statistical analysis

The statistical analysis was conducted using Python Version 3.10 (Python Software Foundation 2024, Welmington, DE, USA) and SPSS 29.0 (SPSS, Armonk, NY, USA). Percentile calculations were performed with the numpy.percentile() function from NumPy [11], while statistical significance was evaluated using the Kolmogorov–Smirnov test implemented in scipy.stats.kstest() from SciPy [12].

### Percentile calculations

Percentile curves for each hematological parameter (leukocytes, thrombocytes, hemoglobin, hematocrit, neutrophilic granulocytes, and lymphocytes) were constructed for the first five postnatal days. Data were stratified by gestational age, with separate calculations for each week from 23 + 0 to 23 + 6 weeks, 24 + 0 to 24 + 6 weeks, and so on, up to 34 + 0 weeks. For each gestational age group and postnatal day, the 10th to 90th percentiles were calculated. These percentiles represent the distribution of values within each group, providing a comprehensive overview of the expected intervals of hematological parameters in preterm neonates during the first week after birth. For the analysis, capillary and arterial blood parameter values were kept together to create the percentiles. As a baseline for future consultation of the data table, we wanted to reduce complexity as much as possible, while also keeping the number of patients per percentile point maximal.

In a sub-analysis, reference intervals (2.5th to 97.5th percentiles) were calculated for the combined gestational age groups of 23 + 0 to 26 + 6 weeks, 27 + 0 to 30 + 6 weeks, and 31 + 0 to 34 + 0 weeks to enhance clinical applicability in a larger cohort with more robust numbers.

In a further sub-analysis, reference intervals (2.5th to 97.5th percentiles) were calculated separately for males and females for the combined gestational age groups of 23 + 0 to 26 + 6 weeks, 27 + 0 to 30 + 6 weeks, and 31 + 0 to 34 + 0 weeks.

### Comparison of hematological parameters in appropriate-for-gestational age and small-for-gestational age neonates

The impact of fetal growth on hematological parameters was investigated by comparing preterm neonates born AGA with those born SGA. Preterm neonates with a birth weight below the 10th centile and/or a fetal diagnosis of IUGR were categorized as SGA neonates. For each gestational age group (23 + 0 to 23 + 6 weeks through 34 + 0 weeks) and each hematological parameter, we compared values obtained between 20 and 36 h after birth. There is a known significant difference between arterial and capillary values [13] that could also be found in our overall data. To accommodate for this discrepancy while also keeping the dataset as large as possible for statistical analysis, we calculated the ratio of arterial and capillary datapoints per week, per blood parameter. The Kolmogorov–Smirnov test was used to assess differences in the distributions of these parameters between the AGA and SGA groups. A *p* value of less than 0.05 was considered statistically significant.

## Results

**Fig. 1:**
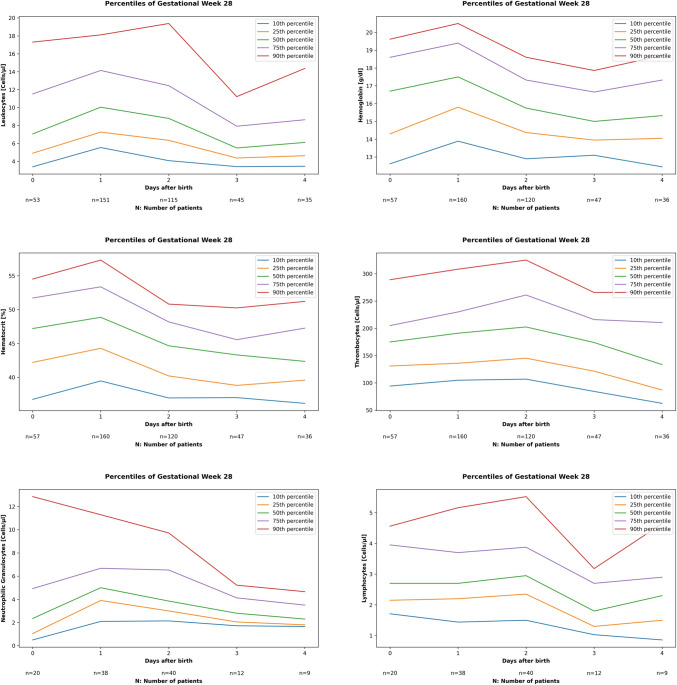
Percentile curves (10th, 25th, 50th, 75th, 90th centile) for preterm neonates born between 28 + 0 and 28 + 6 weeks of gestation for each hematological parameter (leukocytes, thrombocytes, hemoglobin, hematocrit, neutrophilic granulocytes, and lymphocytes) during the first five postnatal days

**Fig. 2:**
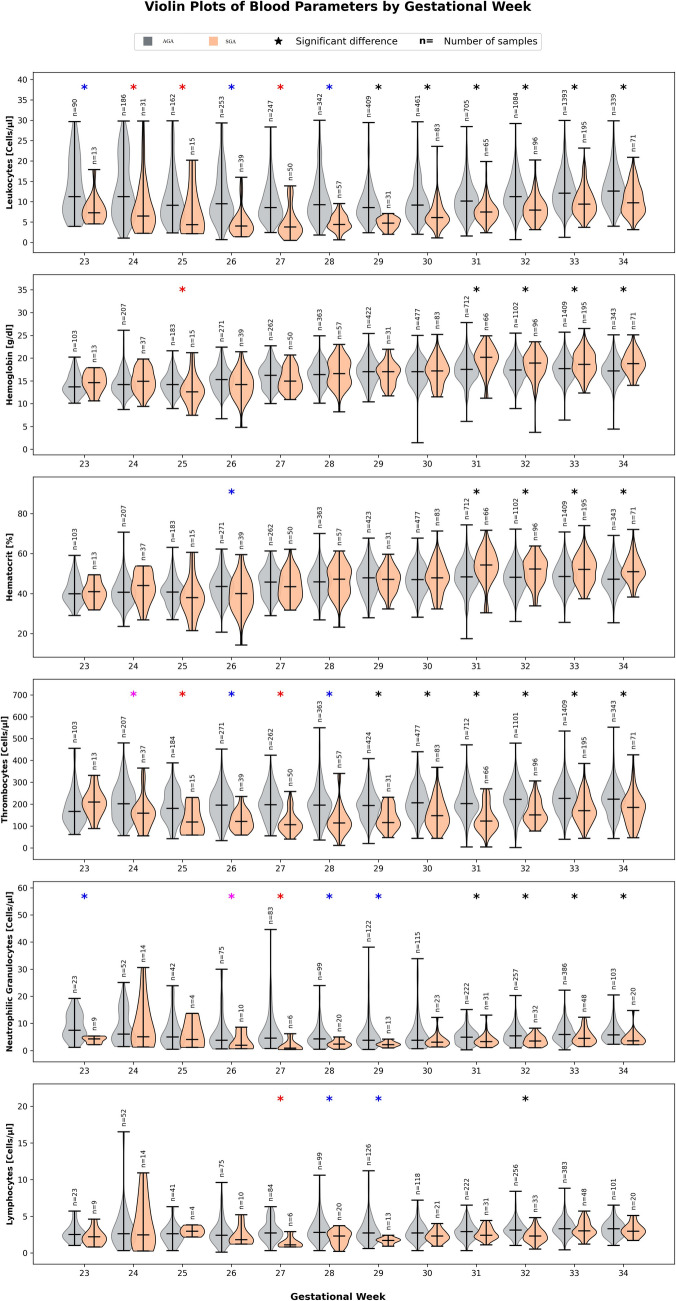
Comparison of hematological parameters (leukocytes, hemoglobin, hematocrit, thrombocytes, neutrophilic granulocytes and lymphocytes) in appropriate-for-gestational age (AGA) and small-for-gestational age (SGA) neonates during the first five days after birth. Black asterisks = significant difference between AGA and SGA, difference in proportion of arterial and capillary sampling technique between < 10%; blue asterisks = significant difference between AGA and SGA, difference in proportion of arterial and capillary sampling technique between 10 and 20%; magenta asterisks = significant difference between AGA and SGA, difference in proportion of arterial and capillary sampling technique between 20 and 30%; red asterisks = significant difference between AGA and SGA, difference in proportion of arterial and capillary sampling technique between 30 and 50%

A total of 3288 preterm neonates ≤ 34 + 0 were born between January 2008 and December 2023 and admitted to the NICU Graz. One-hundred-sixty neonates were excluded because they met one or more of the exclusion criteria listed in Online Supplement Table 1 (Online Supplement Fig. 1, Flow chart). The final study population consisted of 3128 neonates with a median (minimum; maximum) gestational age of 32.1 (23.1;34.0) weeks and a birth weight of 1700 (297; 3210) grams. Demographic data, including causes for preterm birth and short-term outcome parameters, are displayed in Table 1 and Online Supplement Table 2. Specific conditions requiring day-based exclusions related to infection, surgery, medication use, transfusions, or intraventricular hemorrhage. The exact number of included neonates for each parameter for each gestational age is displayed in Figs. 1 and 2 and Table 2.

**Table 1:** Demographic data and outcome parameters of included preterm neonates. Values are presented as n (%), or median (minimum; maximum) as appropriate

	*Entire cohort* *n* = *3128*	*AGA preterm neonates* *n* = *2792*	*SGA preterm neonates* *n* = *328*
*Gestational age* (*weeks*)	32.1 (23.1; 34.0)	32.1 (23.1; 33.9)	32.6 (23.4; 34.0)
*Birth weight* (*g*)	1700 (297; 3210)	1750 (376; 3210)	1240 (297; 1780)
*Female sex n (%)*	1407 (44.8%)	1244 (44.6%)	153 (46.6%)
*Spontaneous vaginal delivery n (%)*	710 (22.7%)	686 (24.6%)	21 (6.4%)
*Apgar 5*	8 (2; 9)	8 (2; 9)	8 (1; 8)
*Umbilical cord pH*	7.31 (7.01; 7.60)	7.31 (7.01; 7.60)	7.29 (7.05; 7.44)
*Prenatal steroids n (%)*	2099 (67.1%)	1886 (67.6%)	208 (63.4%)
*Causes for preterm birth*			
*Preterm rupture of membranes n (%)*	448 (14.3%)	431 (15.4%)	16 (4.9%)
*Amniotic fluid Infection n (%)*	243 (7.8%)	234 (8.4%)	9 (2.7%)
*Gestosis n (%)*	377 (12.1%)	304 (10.9%)	73 (22.3%)
*Cervical insufficiency n (%)*	152 (4.9%)	139 (5.0%)	12 (3.7%)
*Pathological CTG n (%)*	428 (13.7%)	318 (11.4%)	110 (33.5%)
*Preterm labor n (%)*	983 (31.4%)	933 (33.4%)	46 (14.0%)
*Others n (%)*	497 (15.9%)	433 (15.5%)	62 (18.9%)

*AGA* appropriate-for-gestational age, *CTG* cardiotocography, *SGA* small-for-gestational age

**Table 2:** 10th, 25th, 50th, 75th, and 90th percentiles of hematological parameters (leukocytes, thrombocytes, hemoglobin, hematocrit, neutrophilic granulocytes, lymphocytes) for each gestational age (23 + 0 to 34 + 0 weeks) on the first day after birth (comparable to day 1 in Fig. 1). For certain parameters, including lymphocyte counts and neutrophilic granulocytes at the lowest gestational ages, the number of available samples was limited. These percentiles should therefore be interpreted with caution

Gestational age	*n*	10th	25th	50th	75th	90th	n	10th	25th	50th	75th	90th
	**Leukocytes (cells/µL)**						**Thrombocytes (cells/µL)**					
23	30	5023	8943	10,760	19,433	23,023	36	128,000	139,750	177,000	203,000	302,000
24	74	6028	8480	12,940	21,710	25,961	85	124,000	155,000	212,000	264,000	315,800
25	60	5299	7405	9685	14,938	22,623	67	121,400	154,000	190,000	253,000	286,600
26	96	4860	6675	10,485	14,260	20,155	105	105,500	141,000	187,000	246,000	280,800
27	101	4670	7410	10,100	13,205	18,450	107	95,600	124,250	183,000	227,500	285,200
28	151	5540	7260	10,040	14,140	18,110	160	104,900	136,000	191,000	230,000	308,300
29	162	5610	6989	9435	12,150	15,677	168	110,700	138,750	186,500	227,250	282,300
30	205	5836	8020	10,560	14,680	17,410	212	133,100	153,750	193,500	242,250	287,800
31	307	6666	9005	11,520	14,360	17,474	310	111,800	153,250	191,000	238,750	284,100
32	507	7876	9935	12,840	15,775	18,760	518	132,400	166,000	210,000	252,000	292,300
33	691	8700	10790	13,510	16,805	20,270	702	127,000	165,250	211,500	256,000	297,000
34	175	9184	11,190	14,420	17,390	20,494	178	122,000	168,500	212,500	255,000	304,300
	**Hemoglobin (g/dL)**						**Hematocrit (%)**					
23	36	12.8	13.9	15.0	16.8	18.3	36	37.1	40.5	43.1	48.9	50.4
24	85	12.8	13.8	15.2	17.3	18.4	85	37.4	39.4	43.0	48.4	52.2
25	67	12.5	13.7	15.0	16.3	18.4	67	35.0	39.7	42.3	46.5	50.6
26	105	12.9	14.5	16.0	17.3	19.3	105	37.6	40.9	44.6	49.2	54.3
27	107	14.4	15.8	17.3	18.8	20.4	107	40.9	44.2	49.4	52.0	56.8
28	160	13.9	15.8	17.5	19.4	20.5	160	39.5	44.3	48.9	53.4	57.3
29	168	14.7	16.1	17.6	19.2	21.2	168	40.6	44.9	49.1	54.4	58.8
30	212	14.8	16.4	17.9	19.5	20.7	212	41.4	45.6	49.9	54.0	57.5
31	310	15.4	16.8	18.6	20.1	21.2	310	42.9	46.6	51.6	55.1	59.5
32	518	14.7	16.7	18.2	19.8	21.3	518	40.8	45.7	50.0	54.7	58.2
33	702	15.1	16.6	18.3	19.9	21.4	702	41.5	45.8	50.1	54.5	58.9
34	178	14.8	16.3	18.0	19.7	21.0	178	40.8	44.7	49.0	53.9	57.5
	**Neutrophilic granulocytes (cells/µL)**						**Lymphocytes (cells/µL)**					
23	11	2900	4950	7500	14,700	16,700	11	1000	2100	2200	3000	3500
24	24	1920	4175	7500	15,950	20,310	24	1330	2125	2800	4000	6290
25	15	1740	2700	5400	11,850	18,500	15	1520	2225	2700	3250	3760
26	24	1220	2550	4050	6600	15,180	24	845	1538	2200	3300	5170
27	27	3370	4500	5500	10,500	17,700	27	1520	1950	2700	4500	5360
28	38	2095	3900	5000	6675	11,290	38	1440	2200	2700	3700	5160
29	48	2600	3175	4375	6725	15,610	48	1440	1775	2500	3425	5330
30	53	2500	3200	4300	9200	12,420	53	1720	2100	2700	3600	4260
31	98	2670	4125	6050	7550	9430	97	1600	2200	3000	3800	4540
32	114	3100	4575	6200	8575	10,880	114	1930	2600	3100	3900	4770
33	174	4030	5250	7600	10,150	12,300	170	2200	2825	3500	4475	5310
34	41	4200	6200	7800	11,400	14,800	39	2560	2950	3600	4150	4940

### Percentile calculations

Percentile curves, representing the 10th to 90th percentiles, were constructed for each hematological parameter (leukocytes, thrombocytes, hemoglobin, hematocrit, neutrophilic granulocytes, and lymphocytes) during the first five postnatal days. Data were stratified by gestational age in weekly increments, ranging from 23 + 0 to 23 + 6 weeks up to 34 + 0 weeks. To present one example, Fig. 1 displays the percentile curves for preterm neonates born between 28 + 0 and 28 + 6 weeks of gestation for each hematological parameter during the first five postnatal days. Table 2 shows the 10th to 90th percentiles for each parameter and gestational week on the first day after birth. Percentile curves for the other gestational ages are presented in Supplemental A. Although fluctuations in individual parameters were observed across different gestational weeks and postnatal ages, a general trend of increasing values with increasing gestational age was noted.

Supplemental B provides combined reference intervals (2.5 to 97.5 percentile) for hematological parameters, stratified by gestational age at birth (23 + 0 to 26 + 6 weeks, 27 + 0 to 30 + 6 weeks, and 31 + 0 to 34 + 0 weeks). Supplemental C offers the same reference intervals further stratified by sex.

### Comparison of hematological parameters in appropriate-for-gestational age and small-for-gestational age neonates

A total of 2792 preterm neonates were included in the AGA cohort and 328 neonates in the SGA cohort. The mean gestational age and birth weight of neonates in the AGA cohort was median (minimum; maximum) 32.1 (23.1;33.9) weeks of gestation and 1750 (376; 3210) grams, respectively. In the SGA cohort, the median (minimum, maximum) gestational age and birth weight was 32.6 (23.4; 34.0) weeks of gestation and 1240 (297; 1780) grams, respectively. For each gestational age group (23 + 0–23 + 6 weeks through 34 + 0 weeks) and each hematological parameter, values obtained on the first day after birth were compared between the two groups (Fig. 2). Preterm neonates with IUGR/SGA demonstrate distinct hematological profiles compared to AGA preterm neonates, particularly in terms of reduced leukocytes, neutrophilic granulocytes, and thrombocytes, and a trend towards increased hematocrit and hemoglobin. These differences were most consistently observed from 31 gestational weeks onwards, but in part, already seen in younger gestational ages. To exclude a potential bias from varying proportions of capillary versus arterial-derived blood samples, we carefully noted this variation (see color-coded asterisks in Fig. 2).

## Discussion

This study, encompassing a large sample size of very preterm neonates admitted to the NICU Graz between 2008 and 2023, provides robust reference intervals for hematological parameters during the first week after birth. Stringent exclusion criteria ensured a homogenous study population, minimizing the influence of confounders and allowing for a focused analysis of gestational age and postnatal age on hematological parameters. The comprehensive data set, stratified by gestational age in weekly increments, enables clinicians to interpret blood test results with greater precision and identify potential deviations from the established norms. Furthermore, the comparison between SGA and AGA neonates offers valuable insights into the impact of fetal growth on hematological development.

Normal values of complete blood counts in extremely preterm neonates in our cohort differ from previously published data.

Leukocyte counts (50th centile) in our current cohort were lower across all gestational age categories compared to previously reported data [14–17]. In our cohort, the 50th percentile of leukocytes ranged from 7185/µL within the first 24 h after birth in neonates with a gestational age of 25 + 0 to 25 + 6 weeks, to 13,780/µL in neonates with a gestational age of 34 + 0 weeks. In contrast, Scheffer-Mendoza et al. [14] reported higher leukocyte counts, with a median of 17,050/µL at a comparable postnatal age. However, this study [14] focused on full-term neonates delivered vaginally. Overall, these observed differences between preexisting literature and our present study may be attributed to the larger sample size, more detailed stratification by gestational age and postnatal age, and the exclusion of confounding factors in our study. Furthermore, the main difference between our study and the data obtained by, e.g., Scheffer-Mendoza et al. [14] is the gestational age range, as we included preterm neonates from 23 to 34 weeks of gestational age, while Scheffer-Mendoza et al. focused on term neonates. The higher leukocyte values observed in our cohort with more advanced gestational age also highlight the importance of considering gestational age differences. Beside the above-described differences, our findings are largely consistent with the trend of increasing leukocyte counts observed with increasing gestational age, as demonstrated by Wu et al. [18]. Although the absolute values for gestational age categories differed, our study corroborates the general pattern of rising leukocyte counts as gestational age advances. Specifically, the most immature cohort (less than 25 weeks by Wu et al. [18]; 23 + 0 to 23 + 6 weeks in our cohort) exhibited higher leukocyte counts compared to the following week. After this initial peak, a decrease in leukocyte counts was observed, reaching a low point at 29 + 0 to 29 + 6 weeks (Wu et al. [18] and our cohort). Thereafter, a further increase in leukocyte values was seen, with the highest levels occurring at more advanced gestational ages, as mentioned previously.

Comparable to leukocyte counts, lymphocyte values in our cohort are also lower compared to previously reported literature [19]. In our study, the 50th percentile ranged from 2400 lymphocytes/µL in neonates born 23 + 0 to 23 + 6 weeks of gestational age to 4050 lymphocytes/µL, with a gestational age of 34 + 0 weeks. As some gestational age categories included only a small number of neonates, caution is warranted when interpreting these results. Differences were observed when comparing our findings to those of Christensen et al. [19], who reported lymphocytes during the first 3 h after birth, ranging from an estimated 7000/µL in neonates with a gestational age of 23 weeks to 6400/µL in those born at 34 weeks. This discrepancy may be attributed to differences in postnatal age and exclusion criteria. However, our lymphocyte counts were more comparable to the findings of Amatuni et al. [8], who reported counts ranging from 2500/µL in neonates with a gestational age of 22 to 28 weeks to 3450/µL in those born between 32 and 36 weeks.

Our study revealed an initial rise in neutrophilic granulocytes during the first two postnatal days, followed by a decline. This pattern aligns with existing literature, though differences in gestational age must be considered. Prior studies, such as one with a mean gestational age of 38.9 ± 2.4 weeks [20], reported higher absolute values than those in our preterm cohort. For instance, the 25th percentile of neutrophilic granulocytes within the first 24 h in neonates at 34 + 0 weeks was 4560/μL in our data, compared to 6300/μL in term neonates reported by Ianni et al. [17]. This difference was even more notable at the 50th percentile. A potential explanation for this discrepancy is that the study by Ianni et al. included neonates with a mean gestational age of 39.6 ± 1.0 weeks, whereas our analysis focused on extremely and very preterm neonates. Similar trends were observed by Henry and Christensen [9], though their broader inclusion criteria may affect comparability. Despite these differences, our absolute values remain within previously published ranges. A notable similarity with past studies is the low 10th percentile of thrombocyte counts across gestational ages. The lowest 10th percentile value within 24 h post-birth was 85,800/μL in neonates at 29 + 0 to 29 + 6 weeks, comparable to values reported by Henry and Christensen [9]. These counts are well below the adult threshold for thrombocytopenia (150,000/μL) [21], highlighting the need for gestational age-specific reference intervals. While thrombocytopenia may indicate conditions like perinatal asphyxia, sepsis, or NEC [22], our cohort excluded such cases. This strengthens the argument that transient thrombocytopenia may be typical in very preterm neonates, or that current clinical definitions require reassessment.

Hemoglobin and hematocrit values in our cohort of preterm neonates were slightly higher than in term neonates, consistent with previous studies [17]. This is likely due to lower plasma volume and more concentrated erythrocyte levels in preterm neonates [2]. Our results for neonates with a gestational age of 33–35 weeks aligned with Melioli et al. [15], highlighting changes in erythropoiesis that accompany preterm maturation. Hemoglobin remained relatively stable with postnatal age, while hematocrit peaked within the first 24 h across all gestational ages. In neonatal intensive care, red blood transfusions or erythropoietin are commonly used to maintain appropriate hemoglobin and hematocrit levels in preterm infants to prevent anemia. To reduce bias, we excluded factors influencing these values, such as intraventricular hemorrhage, major blood loss, surgery, and transfusion. These exclusions were essential to establish reliable reference intervals that accurately reflect typical hematological profiles in preterm neonates.

Preterm neonates with IUGR/SGA showed notable differences in hematological parameters compared to AGA peers, particularly in leukocytes, thrombocytes, and neutrophilic granulocytes. As Özyürek et al. [23] reported, SGA infants had significantly reduced leukocyte and neutrophil counts through the first week after birth. Similarly, our cohort showed lower thrombocyte counts in SGA neonates—except for those born at 23 + 0 to 23 + 6 weeks. We attribute these reductions to chronic intrauterine stress in SGA cases [24].

Contrary to our expectation that hemoglobin and hematocrit levels might be significantly lower in IUGR/SGA infants due to placental insufficiency and reduced nutrient supply [25], our cohort showed no significant differences between SGA and AGA neonates in these parameters, except for those with a gestational age of 31 + 0 to – 34 + 0 weeks. Lymphocyte differences were only observed in neonates born at 27 + 0 to 27 + 6, 28 + 0 to 28 + 6, 29 + 0 to 29 + 6, and 32 + 0 to 32 + 6 weeks, with lower values in SGA neonates. Özyürek et al. [23] found no differences on day one but did observe them by day seven. Their inclusion of full-term neonates may explain this discrepancy. Our lymphocyte findings should be interpreted with caution due to limited sample size.

### Strength and limitation

This study benefits from a large sample size, with 3175 preterm neonates being eligible for analysis. This allowed us to establish robust reference intervals for hematologic parameters in preterm neonates during the first postnatal week, stratified by gestational and postnatal age. Our strict exclusion criteria led to a relatively homogeneous study population, minimizing potential confounders. Some limitations must be considered. One is the combined use of capillary and arterial blood samples for percentile calculations. A comparison between the two methods was not feasible due to the limited and unevenly distributed data in each group (blood value, gestational age, postnatal age) inherent to the retrospective study design. Figure 2 illustrates the representation of each sampling method by group. Differences between arterial and capillary blood sampling methods have been described in the literature [26]. Capillary and arterial blood differ physiologically and in several pre-analytical aspects (collection technique, sample volume, anticoagulant ratio, risk of hemolysis, tissue fluid contamination), all of which may affect hematologic measurements [27]. These differences are particularly relevant for hemoglobin/hematocrit, leukocyte counts, and platelet values. While reflecting clinical reality—where both sample types depends on patient circumstances—this introduces potential variability. In practice, some neonates have arterial, others capillary values, and it is not feasible to use separate percentiles for each variable. While combining both sources increased sample size and enhanced feasibility, it may also introduce variability in the derived reference intervals. Clinicians should therefore apply these intervals with awareness of sample origin, and future well-powered studies should aim to establish separate, standardized reference ranges for capillary and arterial blood in neonates. The second limitation is the retrospective nature of data collection. Since the data were not prospectively collected and controlled, this may introduce biases and unaccounted confounding factors. It also limits causal interpretations and may affect generalizability. Another limitation is the small sample size in selected gestational subgroups. This particularly affects lymphocyte counts and neutrophilic granulocytes at very low gestational ages, where the robustness of percentiles is weaker. Clinicians should therefore interpret values in these groups with caution. Nonetheless, the large overall sample size and strict exclusion criteria help mitigate some inherent limitations of a retrospective approach. Our findings also emphasize the need for future work comparing standardized reference intervals between arterial and capillary blood samples. To date, no large-scale neonatal study has systematically contrasted these sources. Our data demonstrate feasibility, but further studies with adequate power are needed to generate blood source–specific intervals that can guide clinical care.

## Conclusion

This study provides comprehensive reference intervals for hematological parameters in preterm neonates during the first 5 days after birth, stratified by gestational age and postnatal age.

Our study addresses several previous gaps: prior reference intervals rarely stratified by gestational week, particularly in extremely preterm infants—our data provide week-specific percentiles. Earlier studies generally did not include both arterial and capillary samples—we provide both, while acknowledging variability between them. Moreover, reliable percentile estimates at very low gestational ages were previously lacking; our data now fill this gap, though caution is needed for parameters with limited numbers. By addressing these limitations, our study offers new insights and contributes original reference values for neonatal hematology.

These normative values can guide clinicians in interpreting blood test results, identifying abnormalities, and monitoring the trajectory of hematological parameters in this vulnerable patient population. The observed differences between AGA and SGA preterm neonates warrant further investigation to understand the potential clinical implications of fetal growth restriction on neonatal hematology. The established reference intervals offer valuable clinical utility for assessing hematological status, enabling early identification of potential deviations and informing appropriate interventions. Future research incorporating longitudinal follow-up could elucidate the long-term implications of these early hematological variations and their impact on later health outcomes. Prospective studies controlling for potential confounding factors could strengthen the current findings and enhance their generalizability, ultimately improving clinical decision-making and patient care.

## Supplementary Information

Below is the link to the electronic supplementary material.

Supplementary file1 (DOCX 518 KB)

Supplementary file2 (DOCX 16 KB)

Supplementary file3 (DOCX 21.1 KB)

Supplementary file4 (DOCX 80.0 KB)

Supplementary file5 (DOCX 31 KB)

Supplementary file6 (DOCX 29 KB)

## Data Availability

No datasets were generated or analysed during the current study.

## Abbreviations

IUGR: Intrauterine growth restriction
SGA: Small-for-gestational age
AGA: Appropriate-for-gestational age
SCID: Severe combined immunodeficiency
G-CSF: Granulocyte-colony stimulating factor
