# Supplementary figures and images for: Complete blood count reference intervals for extremely preterm neonates
Source: Eur J Pediatr. 2025 Oct 18;184(11):699. doi: 10.1007/s00431-025-06544-4 (PMC12535521; doi:10.1007/s00431-025-06544-4)

**Online Supplement Figure 1.** Study flow chart


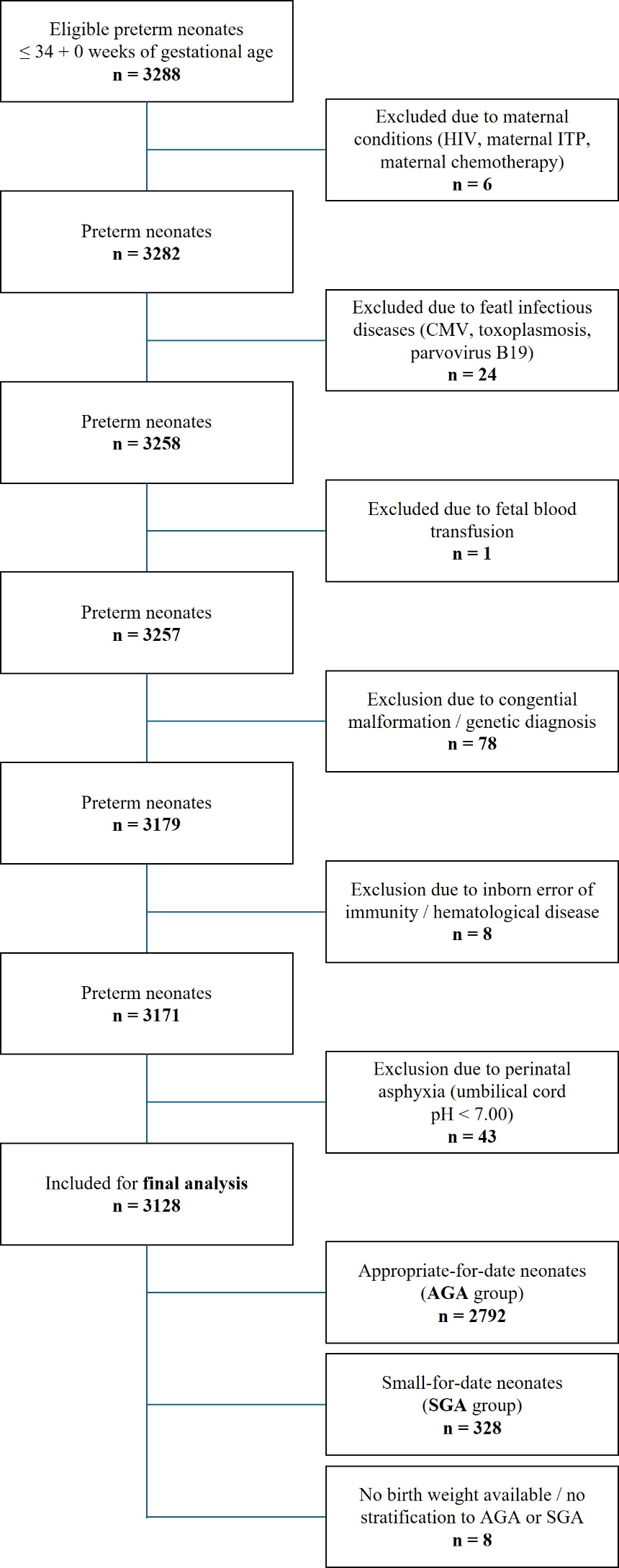

Supplement: Supplementary file 1 — Supplementary file1 (DOCX 518 KB) [file 431_2025_6544_MOESM1_ESM.docx]
