# Supplementary material for: Complete blood count reference intervals for extremely preterm neonates
Source: Eur J Pediatr. 2025 Oct 18;184(11):699. doi: 10.1007/s00431-025-06544-4 (PMC12535521; doi:10.1007/s00431-025-06544-4)
Supplement: Supplementary file 2 — Supplementary file2 (DOCX 16 KB) [file 431_2025_6544_MOESM2_ESM.docx]

**Online Supplement Table 1.** Summary of Exclusion Criteria

| Category | Criteria |
| --- | --- |
| *Neonatal* | Fetal infections (CMV, parvovirus B19, toxoplasmosis) |
|  | Inborn errors of immunity (prenatal or postnatal diagnosis) |
|  | Genetic diseases |
|  | Congenital malformations (severe cardiac, neurological) |
|  | Fetal/umbilical cord transfusions |
|  | Perinatal asphyxia (umbilical cord pH < 7.00) |
|  | Specific medications (corticosteroids, immunoglobulins, G-CSF, filgrastim, indomethacin, ibuprofen, anticonvulsants, erythropoietin^1^, packed red blood cells^1^, platelet transfusions^1^) |
|  | Postnatal events^2^ (fetal inflammatory response syndrome^3^, necrotizing enterocolitis, infection^4^) surgery^5^, intraventricular hemorrhage > grade 2 ^6^ |
| *Maternal* | Immune thrombocytopenia |
|  | Rhesus incompatibility |
|  | Chemotherapy or immunosuppressive therapy during pregnancy |
|  | HIV infection |

***Abbreviation***: CMV = cytomegaly virus, G-CSF = granulocyte-colony stimulating factor (G-CSF), HIV = Human Immunodeficiency Virus

^1^ hemoglobin and hematocrit parameters were excluded after erythropoietin or packed red blood cell administration, and platelet parameters were excluded after platelet transfusions.

^2^ Partial data exclusions from the time of diagnosis

^3^ interleukin-6 ≥ 6 pg/mL in cord blood

^4^ C-reactive protein [CRP] > 10 mg/L and/or leukocyte count > 30,000/µL

^5^ excluding when recovering from surgery (one week post-operatively)

^6^ excluded after the event
