# Supplementary material for: Complete blood count reference intervals for extremely preterm neonates
Source: Eur J Pediatr. 2025 Oct 18;184(11):699. doi: 10.1007/s00431-025-06544-4 (PMC12535521; doi:10.1007/s00431-025-06544-4)
Supplement: Supplementary file 3 — Supplementary file3 (DOCX 21.1 KB) [file 431_2025_6544_MOESM3_ESM.docx]

**Online Supplement Table 2.** Outcome parameters of included preterm neonates. Values are presented as n (%), or median (minimum; maximum) as appropriate.

|  | Entire cohort n=3128 | AGA preterm neonates n=2792 | SGA preterm neonates  n=328 |
| --- | --- | --- | --- |
| IVH any grade n(%) | 273 (8.7%) | 239 (8.6%) | 34 (10.4%) |
| PVH n(%) | 65 (2.1%) | 61 (2.2%) | 4 (1.2%) |
| PVL n(%) | 133 (4.3%) | 126 (4.5%) | 6 (1.8%) |
| LOS n(%) | 224 (7.2%) | 194 (6.9%) | 30 (9.1%) |
| BPD n(%) | 118 (3.8%) | 88 (3.2%) | 30 (9.1%) |
| ROP n(%) | 210 (6.7%) | 175 (6.3%) | 35 (10.7%) |
| NEC n(%) | 60 (1.9%) | 47 (1.7%) | 13 (4.0%) |
| SIP n(%) | 54 (1.7%) | 41 (1.5%) | 13 (4.0%) |
| PDA n(%) | 302 (9.7%) | 266 (9.5%) | 36 (11.0%) |
| Mortality n(%) | 144 (4.6%) | 119 (4.3%) | 24 (7.3%) |

***Abbreviation***: AGA = appropriate-for-gestational age, BPD = bronchopulmonary dysplasia, IVH = intraventricular hemorrhage, LOS = late onset sepsis, NEC = necrotizing enterocolitis, PDA = persistent ductus arteriosus, PVH = periventricular hemorrhage, PVL = periventricular leukomalacia, ROP = retinopathy of prematurity, SGA = small-for-gestational age, SIP = spontaneous intestinal perforation
