# Supplementary material for: Complete blood count reference intervals for extremely preterm neonates
Source: Eur J Pediatr. 2025 Oct 18;184(11):699. doi: 10.1007/s00431-025-06544-4 (PMC12535521; doi:10.1007/s00431-025-06544-4)
Supplement: Supplementary file 4 — Supplementary file4 (DOCX 80.0 KB) [file 431_2025_6544_MOESM4_ESM.docx]

**Supplemental A_1.** 10th, 25th, 50th, 75th, and 90th percentiles of hematological parameters (leukocytes, thrombocytes, hemoglobin, hematocrit, neutrophilic granulocytes, lymphocytes) for each gestational age (23+0 to 34+0 weeks) within the first 24 hours after birth (comparable to day 0 in Figure 1).

|  | **Leukocytes (cells/µL)** | | | | | | **Thrombocytes (cells/µL)** | | | | | |
| --- | --- | --- | --- | --- | --- | --- | --- | --- | --- | --- | --- | --- |
| **Gestational age** | **n** | **10^th^** | **25^th^** | **50^th^** | **75^th^** | **90^th^** | **n** | **10^th^** | **25^th^** | **50^th^** | **75^th^** | **90^th^** |
| **23** | 15 | 4426 | 5150 | 7650 | 11925 | 14782 | 16 | 136500 | 160750 | 216500 | 234750 | 275000 |
| **24** | 36 | 4035 | 5685 | 9570 | 14678 | 21810 | 38 | 133700 | 160750 | 207500 | 255500 | 279500 |
| **25** | 14 | 3512 | 4393 | 7185 | 12320 | 17064 | 17 | 87600 | 131000 | 188000 | 243000 | 274600 |
| **26** | 46 | 4310 | 5690 | 7410 | 98025 | 12765 | 48 | 101550 | 150250 | 185000 | 233000 | 251300 |
| **27** | 101 | 4111 | 5238 | 8065 | 11884 | 13689 | 107 | 73300 | 149000 | 190000 | 232500 | 286200 |
| **28** | 35 | 3390 | 4910 | 7060 | 11520 | 17306 | 36 | 94200 | 131000 | 175000 | 205000 | 289000 |
| **29** | 70 | 4105 | 6390 | 8045 | 11193 | 13459 | 73 | 85800 | 138000 | 184000 | 224000 | 244800 |
| **30** | 46 | 4047 | 5533 | 8085 | 10698 | 14182 | 47 | 107000 | 133000 | 170000 | 207000 | 269000 |
| **31** | 307 | 5740 | 7411 | 9998 | 12213 | 17843 | 310 | 109400 | 142000 | 180000 | 217000 | 271600 |
| **32** | 507 | 6234 | 8200 | 11090 | 14680 | 18096 | 518 | 113400 | 143000 | 196000 | 239000 | 281200 |
| **33** | 691 | 7271 | 9972 | 12840 | 16080 | 20475 | 702 | 115950 | 155750 | 198500 | 241500 | 274100 |
| **34** | 128 | 9200 | 12025 | 13780 | 17103 | 21015 | 129 | 98500 | 133500 | 177000 | 231000 | 261500 |
|  | **Hemoglobin (g/dL)** | | | | | | **Hematocrit (%)** | | | | | |
| **Gestational age** | **n** | **10^th^** | **25^th^** | **50^th^** | **75^th^** | **90^th^** | **n** | **10^th^** | **25^th^** | **50^th^** | **75^th^** | **90^th^** |
| **23** | 16 | 12.8 | 13.8 | 14.8 | 16.1 | 17.5 | 16 | 37.6 | 39.9 | 42.1 | 46.6 | 49.0 |
| **24** | 38 | 12.0 | 13.3 | 14.8 | 16.4 | 19.0 | 38 | 34.7 | 37.9 | 42.8 | 45.6 | 52.6 |
| **25** | 17 | 12.2 | 13.0 | 14.5 | 16.0 | 18.4 | 17 | 34.8 | 37.9 | 41.6 | 44.9 | 52.6 |
| **26** | 48 | 13.4 | 15.1 | 16.3 | 18.9 | 19.9 | 48 | 37.7 | 43.2 | 47.0 | 52.6 | 56.4 |
| **27** | 107 | 13.1 | 15.4 | 17.2 | 18.5 | 20.2 | 107 | 38.1 | 43.9 | 48.7 | 52.1 | 55.6 |
| **28** | 36 | 12.6 | 14.3 | 16.7 | 18.6 | 19.6 | 36 | 36.7 | 42.2 | 47.2 | 51.7 | 54.5 |
| **29** | 73 | 13.4 | 15.9 | 17.2 | 19.2 | 20.6 | 73 | 39.3 | 44.9 | 49.9 | 54.4 | 58.1 |
| **30** | 47 | 14.8 | 15.5 | 17.1 | 18.9 | 20.6 | 47 | 42.0 | 44.7 | 48.3 | 53.6 | 57.3 |
| **31** | 310 | 14.2 | 15.7 | 17.9 | 20.1 | 21.2 | 310 | 40.7 | 44.8 | 51.0 | 56.0 | 59.1 |
| **32** | 518 | 14.1 | 15.9 | 17.9 | 19.7 | 20.9 | 518 | 40.9 | 45.1 | 50.0 | 55.2 | 58.2 |
| **33** | 702 | 14.8 | 16.4 | 18.6 | 20.2 | 22.1 | 702 | 41.6 | 46.1 | 51.9 | 56.0 | 60.7 |
| **34** | 129 | 14.7 | 16.4 | 18.3 | 20.6 | 22.1 | 129 | 42.7 | 47.0 | 51.6 | 57.2 | 63.0 |
|  | **Neutrophilic granulocytes (cells/µL)** | | | | | | **Lymphocytes (cells/µL)** | | | | | |
| **Gestational age** | **n** | **10^th^** | **25^th^** | **50^th^** | **75^th^** | **90^th^** | **n** | **10^th^** | **25^th^** | **50^th^** | **75^th^** | **90^th^** |
| **23** | 6 | 2620 | 3250 | 4650 | 6325 | 7450 | 6 | 2160 | 2250 | 2400 | 2600 | 2780 |
| **24** | 17 | 2700 | 2950 | 4200 | 16700 | 1800 | 17 | 1000 | 1550 | 1900 | 2250 | 2400 |
| **25** | 15 | 1407 | 2900 | 4650 | 7625 | 12150 | 15 | 1530 | 1750 | 2400 | 2775 | 3480 |
| **26** | 18 | 2010 | 3100 | 3400 | 7425 | 18870 | 18 | 2010 | 2325 | 2650 | 3275 | 3730 |
| **27** | 18 | 2200 | 2900 | 4350 | 7175 | 10750 | 18 | 1180 | 2075 | 2750 | 3150 | 3950 |
| **28** | 40 | 500 | 1050 | 2350 | 4925 | 12850 | 40 | 1710 | 2150 | 2700 | 3950 | 4560 |
| **29** | 48 | 1310 | 1975 | 3500 | 6300 | 11080 | 48 | 1700 | 2175 | 2850 | 4050 | 5290 |
| **30** | 11 | 2400 | 3200 | 3900 | 6700 | 7800 | 12 | 1280 | 2000 | 2350 | 3500 | 5330 |
| **31** | 98 | 1050 | 1800 | 3550 | 5550 | 9870 | 97 | 1800 | 2175 | 3050 | 3450 | 4460 |
| **32** | 32 | 1300 | 1700 | 6150 | 7400 | 11460 | 31 | 2040 | 2400 | 3100 | 4600 | 5300 |
| **33** | 140 | 2450 | 4625 | 6900 | 9775 | 12050 | 141 | 2100 | 2825 | 3800 | 4400 | 4600 |
| **34** | 9 | 4560 | 5175 | 6450 | 7425 | 7890 | 9 | 2470 | 3250 | 4050 | 4625 | 4850 |

**Supplemental A_2.** 10th, 25th, 50th, 75th, and 90th percentiles of hematological parameters (leukocytes, thrombocytes, hemoglobin, hematocrit, neutrophilic granulocytes, lymphocytes) for each gestational age (23+0 to 34+0 weeks) on the second day after birth (comparable to day 2 in Figure 1).

|  | **Leukocytes (cells/µL)** | | | | | | **Thrombocytes (cells/µL)** | | | | | |
| --- | --- | --- | --- | --- | --- | --- | --- | --- | --- | --- | --- | --- |
| **Gestational age** | **n** | **10^th^** | **25^th^** | **50^th^** | **75^th^** | **90^th^** | **n** | **10^th^** | **25^th^** | **50^th^** | **75^th^** | **90^th^** |
| **23** | 27 | 6426 | 8270 | 12370 | 20465 | 24216 | 32 | 94200 | 129750 | 181000 | 253500 | 304000 |
| **24** | 52 | 5201 | 6555 | 10775 | 19288 | 24433 | 62 | 112100 | 140250 | 201500 | 280250 | 347300 |
| **25** | 60 | 4979 | 6025 | 8860 | 11888 | 16314 | 67 | 105500 | 135500 | 173500 | 223250 | 256250 |
| **26** | 76 | 3000 | 6098 | 8690 | 16303 | 19730 | 78 | 98700 | 134000 | 186500 | 255250 | 299500 |
| **27** | 44 | 3774 | 5658 | 7965 | 10270 | 14074 | 45 | 96800 | 139000 | 192000 | 224000 | 272200 |
| **28** | 53 | 4078 | 6345 | 8790 | 12445 | 19380 | 57 | 106900 | 145250 | 202500 | 261000 | 325000 |
| **29** | 54 | 4932 | 6100 | 8250 | 11035 | 15198 | 55 | 115700 | 151250 | 203250 | 247000 | 295400 |
| **30** | 70 | 4875 | 6200 | 8320 | 11018 | 14280 | 71 | 119000 | 156000 | 208000 | 264500 | 300000 |
| **31** | 76 | 5510 | 7243 | 9120 | 11553 | 14165 | 77 | 130000 | 171000 | 214000 | 256500 | 309300 |
| **32** | 360 | 6622 | 8560 | 10155 | 12518 | 15458 | 362 | 135400 | 180750 | 230500 | 281750 | 332700 |
| **33** | 76 | 6940 | 8315 | 10360 | 12920 | 16072 | 77 | 153000 | 188000 | 238000 | 283000 | 319000 |
| **34** | 175 | 6917 | 8882 | 11100 | 13378 | 16231 | 178 | 151800 | 187000 | 225000 | 277000 | 317000 |
|  | **Hemoglobin (g/dL)** | | | | | | **Hematocrit (%)** | | | | | |
| **Gestational age** | **n** | **10^th^** | **25^th^** | **50^th^** | **75^th^** | **90^th^** | **n** | **10^th^** | **25^th^** | **50^th^** | **75^th^** | **90^th^** |
| **23** | 32 | 11.8 | 12.4 | 13.0 | 14.8 | 17.4 | 32 | 34.2 | 35.7 | 38.6 | 42.7 | 49.0 |
| **24** | 62 | 11.9 | 12.3 | 13.5 | 15.1 | 16.6 | 62 | 33.9 | 35.6 | 39.5 | 44.3 | 48.8 |
| **25** | 67 | 11.5 | 12.3 | 13.8 | 15.0 | 17.2 | 67 | 33.0 | 35.2 | 40.4 | 43.4 | 49.4 |
| **26** | 78 | 11.5 | 12.6 | 14.1 | 15.8 | 17.4 | 78 | 33.5 | 36.0 | 40.0 | 44.5 | 50.1 |
| **27** | 45 | 12.8 | 13.7 | 15.2 | 16.9 | 18.1 | 45 | 36.5 | 39.1 | 43.7 | 47.3 | 49.8 |
| **28** | 57 | 12.9 | 14.4 | 15.8 | 17.3 | 18.6 | 57 | 37.0 | 40.2 | 44.7 | 48.2 | 50.8 |
| **29** | 54 | 13.4 | 15.0 | 16.6 | 18.2 | 19.4 | 54 | 38.2 | 41.0 | 45.9 | 51.0 | 53.7 |
| **30** | 71 | 13.5 | 14.6 | 16.6 | 18.5 | 19.8 | 71 | 37.7 | 40.9 | 45.7 | 50.4 | 54.9 |
| **31** | 77 | 14.2 | 15.6 | 17.1 | 18.7 | 20.7 | 77 | 39.3 | 42.9 | 47.1 | 51.3 | 57.2 |
| **32** | 363 | 13.8 | 15.4 | 16.9 | 18.6 | 20.0 | 363 | 38.7 | 42.3 | 46.5 | 51.1 | 54.9 |
| **33** | 77 | 14.4 | 15.6 | 17.3 | 18.7 | 20.5 | 77 | 39.7 | 43.0 | 47.2 | 51.6 | 56.4 |
| **34** | 178 | 14.2 | 15.7 | 16.9 | 18.3 | 20.0 | 178 | 39.7 | 43.2 | 46.3 | 50.3 | 55.3 |
|  | **Neutrophilic granulocytes (cells/µL)** | | | | | | **Lymphocytes (cells/µL)** | | | | | |
| **Gestational age** | **n** | **10^th^** | **25^th^** | **50^th^** | **75^th^** | **90^th^** | **n** | **10^th^** | **25^th^** | **50^th^** | **75^th^** | **90^th^** |
| **23** | 8 | 3530 | 4250 | 5350 | 7300 | 12010 | 8 | 1870 | 2125 | 2700 | 3375 | 4320 |
| **24** | 8 | 3120 | 4500 | 5600 | 8000 | 15560 | 8 | 1100 | 1500 | 2300 | 4500 | 6600 |
| **25** | 12 | 2170 | 2875 | 4550 | 7575 | 10770 | 11 | 900 | 1300 | 2700 | 3100 | 3600 |
| **26** | 18 | 1150 | 2675 | 3400 | 4625 | 6360 | 18 | 910 | 1500 | 1850 | 2575 | 3160 |
| **27** | 3 | 1800 | 2450 | 3800 | 8150 | 14300 | 3 | 1400 | 1800 | 2600 | 3350 | 4800 |
| **28** | 9 | 2140 | 3000 | 3850 | 6525 | 9720 | 9 | 1500 | 2350 | 2950 | 3875 | 5520 |
| **29** | 32 | 2030 | 2500 | 4000 | 8700 | 15320 | 32 | 1500 | 1800 | 2700 | 3900 | 4780 |
| **30** | 53 | 1850 | 2400 | 3350 | 7050 | 11950 | 53 | 1500 | 1825 | 2400 | 3375 | 4200 |
| **31** | 28 | 1700 | 2700 | 4500 | 6300 | 7900 | 28 | 1800 | 2200 | 2750 | 3475 | 4320 |
| **32** | 22 | 2500 | 3200 | 4400 | 6325 | 7550 | 22 | 1900 | 2500 | 3100 | 3700 | 4240 |
| **33** | 2 | 2700 | 3675 | 4850 | 6700 | 8700 | 2 | 2200 | 2600 | 3100 | 3800 | 4600 |
| **34** | 45 | 2940 | 3700 | 5200 | 6500 | 8160 | 45 | 1980 | 2300 | 3000 | 3700 | 4400 |

**Supplemental A_3.** 10th, 25th, 50th, 75th, and 90th percentiles of hematological parameters (leukocytes, thrombocytes, hemoglobin, hematocrit, neutrophilic granulocytes, lymphocytes) for each gestational age (23+0 to 34+0 weeks) on the third day after birth (comparable to day 3 in Figure 1).

|  | **Leukocytes (cells/µL)** | | | | | | **Thrombocytes (cells/µL)** | | | | | |
| --- | --- | --- | --- | --- | --- | --- | --- | --- | --- | --- | --- | --- |
| **Gestational age** | **n** | **10^th^** | **25^th^** | **50^th^** | **75^th^** | **90^th^** | **n** | **10^th^** | **25^th^** | **50^th^** | **75^th^** | **90^th^** |
| **23** | 15 | 5960 | 6933 | 9690 | 16993 | 23152 | 16 | 73250 | 93000 | 136000 | 185750 | 236000 |
| **24** | 32 | 4173 | 5363 | 7570 | 11825 | 20575 | 34 | 69000 | 101750 | 170000 | 195000 | 246900 |
| **25** | 42 | 4168 | 5480 | 9540 | 16291 | 20156 | 45 | 69000 | 114750 | 151000 | 218000 | 295800 |
| **26** | 38 | 2631 | 3859 | 7070 | 10663 | 14247 | 42 | 59200 | 108250 | 172000 | 222750 | 311400 |
| **27** | 30 | 2108 | 4548 | 6155 | 8190 | 13612 | 31 | 84400 | 111000 | 189000 | 265000 | 287200 |
| **28** | 115 | 3410 | 4370 | 5490 | 7920 | 11220 | 120 | 84400 | 121500 | 174000 | 216000 | 265600 |
| **29** | 127 | 3588 | 4675 | 6090 | 8403 | 9567 | 130 | 108200 | 151500 | 206000 | 239000 | 279800 |
| **30** | 146 | 4286 | 5340 | 6690 | 8740 | 12022 | 151 | 99200 | 157500 | 219000 | 283500 | 310000 |
| **31** | 236 | 4990 | 6215 | 7530 | 9580 | 12110 | 238 | 110500 | 170750 | 215500 | 269250 | 306000 |
| **32** | 121 | 5300 | 6620 | 8250 | 10380 | 11990 | 122 | 122500 | 185250 | 235500 | 289500 | 312800 |
| **33** | 156 | 5770 | 6923 | 8815 | 10938 | 13620 | 156 | 129000 | 175000 | 235500 | 303500 | 357000 |
| **34** | 37 | 5882 | 6730 | 8680 | 10070 | 12438 | 37 | 103800 | 191000 | 221000 | 259000 | 296400 |
|  | **Hemoglobin (g/dL)** | | | | | | **Hematocrit (%)** | | | | | |
| **Gestational age** | **n** | **10^th^** | **25^th^** | **50^th^** | **75^th^** | **90^th^** | **n** | **10^th^** | **25^th^** | **50^th^** | **75^th^** | **90^th^** |
| **23** | 16 | 10.9 | 11.4 | 12.8 | 13.7 | 16.3 | 16 | 31.8 | 35.0 | 37.8 | 40.1 | 44.2 |
| **24** | 34 | 10.5 | 11.6 | 12.7 | 14.2 | 17.2 | 34 | 29.2 | 32.3 | 37.2 | 41.1 | 47.6 |
| **25** | 45 | 10.7 | 11.1 | 12.4 | 13.6 | 14.3 | 45 | 29.4 | 32.2 | 35.2 | 39.1 | 41.4 |
| **26** | 42 | 10.9 | 11.9 | 13.9 | 15.8 | 17.0 | 42 | 31.1 | 33.2 | 40.3 | 45.0 | 47.8 |
| **27** | 31 | 12.3 | 12.8 | 14.1 | 15.6 | 17.8 | 31 | 34.9 | 37.2 | 40.0 | 43.6 | 48.7 |
| **28** | 120 | 13.1 | 14.0 | 15.0 | 16.7 | 17.9 | 120 | 37.0 | 38.8 | 43.3 | 45.6 | 50.2 |
| **29** | 129 | 12.1 | 13.3 | 15.9 | 17.6 | 19.5 | 130 | 35.1 | 37.5 | 44.6 | 49.9 | 54.1 |
| **30** | 151 | 12.7 | 14.0 | 15.4 | 17.5 | 19.2 | 151 | 34.6 | 38.6 | 43.7 | 48.1 | 53.3 |
| **31** | 238 | 13.6 | 15.4 | 17.4 | 19.4 | 20.9 | 238 | 37.9 | 42.4 | 48.2 | 52.6 | 56.9 |
| **32** | 122 | 13.3 | 14.7 | 16.4 | 18.3 | 19.7 | 122 | 37.1 | 41.0 | 45.9 | 50.6 | 54.3 |
| **33** | 156 | 14.1 | 15.5 | 16.8 | 18.2 | 20.4 | 156 | 38.9 | 42.8 | 46.0 | 50.8 | 55.2 |
| **34** | 37 | 13.3 | 14.8 | 16.9 | 18.7 | 19.4 | 37 | 37.3 | 42.2 | 46.9 | 50.0 | 53.1 |
|  | **Neutrophilic granulocytes (cells/µL)** | | | | | | **Lymphocytes (cells/µL)** | | | | | |
| **Gestational age** | **n** | **10^th^** | **25^th^** | **50^th^** | **75^th^** | **90^th^** | **n** | **10^th^** | **25^th^** | **50^th^** | **75^th^** | **90^th^** |
| **23** | 3 | 4225 | 5250 | 5850 | 9750 | 11950 | 3 | 1350 | 1500 | 1525 | 2113 | 3950 |
| **24** | 11 | 2900 | 5075 | 8800 | 12400 | 14110 | 11 | 2320 | 2950 | 3550 | 5400 | 6660 |
| **25** | 4 | 3860 | 4400 | 5750 | 8400 | 11280 | 4 | 970 | 1375 | 2550 | 4200 | 5460 |
| **26** | 24 | 1700 | 1900 | 2700 | 8150 | 16100 | 24 | 1100 | 1250 | 1800 | 3200 | 4800 |
| **27** | 27 | 1800 | 2400 | 3100 | 4100 | 8500 | 27 | 2000 | 2250 | 2700 | 4100 | 5400 |
| **28** | 12 | 1720 | 2050 | 2800 | 4125 | 5220 | 12 | 1030 | 1300 | 1800 | 2700 | 3180 |
| **29** | 37 | 1200 | 2200 | 3000 | 3700 | 6180 | 37 | 1400 | 1750 | 2000 | 2450 | 3450 |
| **30** | 46 | 1010 | 1375 | 2500 | 4125 | 10020 | 46 | 1680 | 1900 | 3000 | 3700 | 3960 |
| **31** | 13 | 1620 | 2275 | 2950 | 4200 | 5670 | 13 | 1720 | 2100 | 2850 | 3625 | 3990 |
| **32** | 96 | 1800 | 2700 | 3450 | 5325 | 7230 | 97 | 1900 | 2200 | 2800 | 3300 | 4100 |
| **33** | 42 | 1820 | 2400 | 3750 | 5325 | 6350 | 42 | 1555 | 2100 | 2750 | 3375 | 4170 |
| **34** | 41 | 2560 | 2700 | 2900 | 3400 | 4450 | 39 | 1660 | 1900 | 2400 | 3600 | 4240 |

**Supplemental A_4.** 10th, 25th, 50th, 75th, and 90th percentiles of hematological parameters (leukocytes, thrombocytes, hemoglobin, hematocrit, neutrophilic granulocytes, lymphocytes) for each gestational age (23+0 to 34+0 weeks) on the fourth day after birth (comparable to day 4 in Figure 1).

|  | **Leukocytes (cells/µL)** | | | | | | **Thrombocytes (cells/µL)** | | | | | |
| --- | --- | --- | --- | --- | --- | --- | --- | --- | --- | --- | --- | --- |
| **Gestational age** | **n** | **10^th^** | **25^th^** | **50^th^** | **75^th^** | **90^th^** | **n** | **10^th^** | **25^th^** | **50^th^** | **75^th^** | **90^th^** |
| **23** | 16 | 6135 | 7020 | 9355 | 19235 | 21875 | 16 | 79000 | 106750 | 140000 | 212500 | 230000 |
| **24** | 23 | 3891 | 4830 | 7270 | 12535 | 17376 | 25 | 81200 | 105000 | 131000 | 171000 | 306600 |
| **25** | 39 | 3943 | 5180 | 6490 | 7265 | 12559 | 46 | 95400 | 138000 | 177000 | 266000 | 271400 |
| **26** | 33 | 2912 | 4680 | 8200 | 16470 | 20518 | 34 | 77650 | 112375 | 161000 | 239000 | 260900 |
| **27** | 2 | 3400 | 4818 | 5950 | 10123 | 12369 | 2 | 105000 | 123000 | 190000 | 249500 | 293000 |
| **28** | 45 | 3452 | 4630 | 6110 | 8640 | 14364 | 47 | 62500 | 87000 | 133500 | 210500 | 266500 |
| **29** | 27 | 3740 | 4750 | 5650 | 7605 | 9912 | 29 | 90800 | 122000 | 153000 | 207000 | 270200 |
| **30** | 73 | 4495 | 5320 | 6770 | 9115 | 12535 | 75 | 100000 | 132000 | 202000 | 261000 | 311200 |
| **31** | 55 | 4328 | 5200 | 6860 | 8645 | 11068 | 56 | 103500 | 154750 | 211500 | 308250 | 340000 |
| **32** | 67 | 4486 | 5450 | 7530 | 9345 | 12394 | 68 | 103050 | 143750 | 203500 | 286500 | 334100 |
| **33** | 459 | 5705 | 7220 | 8050 | 10455 | 14148 | 461 | 117800 | 165000 | 238000 | 303000 | 344000 |
| **34** | 20 | 6584 | 7168 | 8240 | 10223 | 11102 | 20 | 109100 | 133750 | 204000 | 285250 | 325000 |
|  | **Hemoglobin (g/dL)** | | | | | | **Hematocrit (%)** | | | | | |
| **Gestational age** | **n** | **10^th^** | **25^th^** | **50^th^** | **75^th^** | **90^th^** | **n** | **10^th^** | **25^th^** | **50^th^** | **75^th^** | **90^th^** |
| **23** | 16 | 10.7 | 11.8 | 12.8 | 14.2 | 14.9 | 16 | 30.6 | 33.9 | 37.4 | 39.8 | 42.6 |
| **24** | 25 | 11.0 | 11.8 | 12.6 | 14.5 | 14.8 | 25 | 31.1 | 34.0 | 35.9 | 40.6 | 42.7 |
| **25** | 45 | 11.4 | 11.9 | 13.2 | 14.4 | 14.9 | 45 | 32.9 | 35.4 | 39.0 | 40.6 | 42.6 |
| **26** | 34 | 11.2 | 12.4 | 13.8 | 15.8 | 17.5 | 34 | 32.1 | 34.4 | 39.1 | 44.6 | 48.6 |
| **27** | 2 | 11.3 | 12.8 | 13.6 | 16.6 | 17.7 | 2 | 33.3 | 35.6 | 38.8 | 45.8 | 48.3 |
| **28** | 47 | 12.5 | 14.1 | 15.3 | 17.3 | 18.7 | 47 | 36.2 | 39.6 | 42.4 | 47.3 | 51.2 |
| **29** | 29 | 11.2 | 13.4 | 15.3 | 17.2 | 18.7 | 29 | 31.9 | 37.6 | 44.1 | 49.1 | 51.3 |
| **30** | 75 | 12.3 | 14.0 | 15.9 | 17.5 | 19.2 | 75 | 34.1 | 39.7 | 43.3 | 47.0 | 52.2 |
| **31** | 56 | 12.7 | 14.4 | 16.3 | 17.6 | 19.7 | 56 | 33.9 | 39.7 | 45.5 | 49.0 | 54.6 |
| **32** | 68 | 14.2 | 15.1 | 16.2 | 17.8 | 19.7 | 68 | 39.2 | 41.7 | 45.1 | 48.9 | 54.4 |
| **33** | 461 | 14.4 | 15.7 | 17.1 | 19.2 | 20.6 | 461 | 39.6 | 43.3 | 47.3 | 53.5 | 55.9 |
| **34** | 20 | 14.7 | 15.6 | 18.6 | 19.2 | 20.5 | 20 | 40.7 | 43.2 | 49.9 | 52.2 | 55.2 |
|  | **Neutrophilic granulocytes (cells/µL)** | | | | | | **Lymphocytes (cells/µL)** | | | | | |
| **Gestational age** | **n** | **10^th^** | **25^th^** | **50^th^** | **75^th^** | **90^th^** | **n** | **10^th^** | **25^th^** | **50^th^** | **75^th^** | **90^th^** |
| **23** | 4 | 5220 | 5250 | 530 | 9550 | 12100 | 4 | 1260 | 1650 | 2300 | 4000 | 5020 |
| **24** | 6 | 1500 | 1750 | 4250 | 6225 | 9200 | 6 | 1050 | 1230 | 1717 | 3150 | 4450 |
| **25** | 1 | 3400 | 3400 | 3400 | 3400 | 3400 |  |  |  |  |  |  |
| **26** | 11 | 1000 | 1700 | 2100 | 9050 | 12000 | 1 | 1700 | 2100 | 2500 | 3300 | 4800 |
| **27** | 11 | 1000 | 1600 | 2600 | 8150 | 10080 | 11 | 1160 | 1400 | 1800 | 2400 | 2760 |
| **28** | 38 | 1660 | 1800 | 2300 | 3500 | 4660 | 38 | 0860 | 1500 | 2300 | 2900 | 4640 |
| **29** | 6 | 1000 | 1000 | 1600 | 4225 | 7375 | 6 | 0800 | 1225 | 2050 | 3100 | 6175 |
| **30** | 18 | 1300 | 1700 | 2300 | 2700 | 3600 | 19 | 1240 | 1600 | 2300 | 2700 | 4540 |
| **31** | 32 | 1080 | 2000 | 2300 | 3000 | 4580 | 32 | 1340 | 2200 | 2700 | 2900 | 3360 |
| **32** | 1 | 1420 | 1850 | 2700 | 3925 | 4680 | 1 | 1220 | 2200 | 2525 | 3375 | 3880 |
| **33** | 32 | 1900 | 2525 | 3000 | 3725 | 8540 | 32 | 1920 | 2400 | 3250 | 3825 | 4380 |
| **34** | 17 | 2480 | 3700 | 4100 | 5900 | 6000 | 17 | 1880 | 2500 | 3000 | 3100 | 3320 |
