# Supplementary material for: Complete blood count reference intervals for extremely preterm neonates
Source: Eur J Pediatr. 2025 Oct 18;184(11):699. doi: 10.1007/s00431-025-06544-4 (PMC12535521; doi:10.1007/s00431-025-06544-4)
Supplement: Supplementary file 5 — Supplementary file5 (DOCX 31 KB) [file 431_2025_6544_MOESM5_ESM.docx]

**Supplemental B_1.** 2.5th, 50^th^ and 97.5^th^ percentiles of hematological parameters (leukocytes, thrombocytes, hemoglobin, hematocrit, neutrophilic granulocytes, lymphocytes) for combined gestational age groups of 23+0 to 26+6 weeks, 27+0 to 30+6 weeks, and 31+0 to 34+0 weeks, on the first day after birth.

|  | **Leukocytes (cells/µL)** | | | | **Thrombocytes (cells/µL)** | | | |
| --- | --- | --- | --- | --- | --- | --- | --- | --- |
| **Gestational age** | **n** | **2.5^th^** | **50^th^** | **97.5^th^** | **n** | **2.5^th^** | **50^th^** | **97.5^th^** |
| **23+0 to 26+6** | 260 | 2886 | 11050 | 28549 | 293 | 83050 | 193000 | 361400 |
| **27+0 to 30+6** | 619 | 3544 | 10110 | 25008 | 647 | 79150 | 189000 | 339400 |
| **31+0 to 34+0** | 1680 | 6029 | 12960 | 24030 | 1708 | 93838 | 207000 | 344650 |
|  | **Hemoglobin (g/dL)** | | | | **Hematocrit (%)** | | | |
| **Gestational age** | **n** | **2.5^th^** | **50^th^** | **97.5^th^** | **n** | **2.5^th^** | **50^th^** | **97.5^th^** |
| **23+0 to 26+6** | 293 | 11.5 | 15.4 | 21.0 | 293 | 32.6 | 43.8 | 57.8 |
| **27+0 to 30+6** | 647 | 13.0 | 17.7 | 22.3 | 647 | 36.1 | 49.4 | 62.4 |
| **31+0 to 34+0** | 1708 | 13.5 | 18.3 | 23.0 | 1708 | 37.3 | 50.2 | 62.8 |
|  | **Neutrophilic granulocytes (cells/µL)** | | | | **Lymphocytes (cells/µL)** | | | |
| **Gestational age** | **n** | **2.5^th^** | **50^th^** | **97.5^th^** | **n** | **2.5^th^** | **50^th^** | **97.5^th^** |
| **23+0 to 26+6** | 74 | 1030 | 5400 | 25590 | 74 | 291 | 2600 | 8178 |
| **27+0 to 30+6** | 166 | 1456 | 4900 | 28300 | 166 | 1113 | 2700 | 6288 |
| **31+0 to 34+0** | 427 | 2265 | 6800 | 15100 | 420 | 1500 | 3300 | 6200 |

**Supplemental B_2.** This table presents the statistical comparison for the 2.5th, 50^th^ and 97.5^th^ percentiles of hematological parameters (leukocytes, thrombocytes, hemoglobin, hematocrit, neutrophilic granulocytes, lymphocytes) between two defined interval groups. It includes the calculated Z-score, the corresponding critical value, and an evaluation of whether the Z-score exceeds the critical threshold. Additionally, a condition related to the statistical model is assessed. The table also provides p-values derived from normal distribution calculations, which indicate the significance of the observed differences.

|  | **Leukocytes (cells/µL)** | | | | | | **Thrombocytes (cells/µL)** | | | | | |
| --- | --- | --- | --- | --- | --- | --- | --- | --- | --- | --- | --- | --- |
| **Gestational age cohort** | **Z-score** | **critical value** | **Z>Z_crit​_** | **s2​/(s2​−s1​) <3** | **p-value 1** | **p-value 2** | **Z-score** | **critical value** | **Z>Z_crit​_** | **s2​/(s2​−s1​) <3** | **p-value 1** | **p-value 2** |
| **23+0 to 26+6 vs. 27+0 to 30+6** | 3.58 | 5.74 | False | True | < 0.001 | < 0.001 | 1.71 | 5.94 | False | True | 0.104 | 0.0613 |
| **23+0 to 26+6 vs. 31+0 to 34+0** | 1.61 | 8.53 | False | True | < 0.001 | < 0.001 | 1.73 | 8.66 | False | True | 0.104 | 0.354 |
| **27+0 to 30+6 vs. 31+0 to 34+0** | 10.31 | 9.29 | True | True | < 0.001 | < 0.001 | 5.24 | 9.40 | False | True | 0.061 | 0.354 |
|  | **Hemoglobin (g/dL)** | | | | | | **Hematocrit (%)** | | | | | |
| **Gestational age cohort** | **Z-score** | **critical value** | **Z>Z_crit​_** | **s2​/(s2​−s1​) <3** | **p-value 1** | **p-value 2** | **Z-score** | **critical value** | **Z>Z_crit​_** | **s2​/(s2​−s1​) <3** | **p-value 1** | **p-value 2** |
| **23+0 to 26+6 vs. 27+0 to 30+6** | 12.16 | 5.94 | True | True | 0.561 | 0.928 | 10.47 | 5.94 | True | True | 0.445 | 0.868 |
| **23+0 to 26+6 vs. 31+0 to 34+0** | 17.55 | 8.66 | True | True | 0.561 | 0.565 | 14.27 | 8.66 | True | True | 0.445 | 0.919 |
| **27+0 to 30+6 vs. 31+0 to 34+0** | 5.33 | 9.40 | False | True | 0.928 | 0.565 | 3.48 | 9.40 | False | true | 0.868 | 0.919 |
|  | **Neutrophilic granulocytes (cells/µL)** | | | | | | **Lymphocytes (cells/µL)** | | | | | |
| **Gestational age cohort** | **Z-score** | **critical value** | **Z>Z_crit​_** | **s2​/(s2​−s1​) <3** | **p-value 1** | **p-value 2** | **Z-score** | **critical value** | **Z>Z_crit​_** | **s2​/(s2​−s1​) <3** | **p-value 1** | **p-value 2** |
| **23+0 to 26+6 vs. 27+0 to 30+6** | 0.94 | 3.00 | False | True | 0.005 | <0.001 | 0.05 | 3.00 | False | True | 0.006 | 0.010 |
| **23+0 to 26+6 vs. 31+0 to 34+0** | 122 | 4.33 | False | True | 0.004 | 0.011 | 1.47 | 4.30 | False | true | 0.006 | 0.017 |
| **27+0 to 30+6 vs. 31+0 to 34+0** | 005 | 4.72 | False | True | <0.001 | 0.011 | 3.20 | 4.69 | False | True | 0.01 | 0.017 |
