# Supplementary material for: Complete blood count reference intervals for extremely preterm neonates
Source: Eur J Pediatr. 2025 Oct 18;184(11):699. doi: 10.1007/s00431-025-06544-4 (PMC12535521; doi:10.1007/s00431-025-06544-4)
Supplement: Supplementary file 6 — Supplementary file6 (DOCX 29 KB) [file 431_2025_6544_MOESM6_ESM.docx]

**Supplemental C_1.** 2.5th, 50^th^ and 97.5^th^ percentiles of hematological parameters (leukocytes, thrombocytes, hemoglobin, hematocrit, neutrophilic granulocytes, lymphocytes) for female preterm neonates, stratified in gestational age groups of 23+0 to 26+6 weeks, 27+0 to 30+6 weeks, and 31+0 to 34+0 weeks, on the first day after birth.

|  | **Leukocytes (cells/µL)** | | | | **Thrombocytes (cells/µL)** | | | |
| --- | --- | --- | --- | --- | --- | --- | --- | --- |
| **Gestational age** | **n** | **2.5^th^** | **50^th^** | **97.5^th^** | **n** | **2.5^th^** | **50^th^** | **97.5^th^** |
| **23+0 to 26+6** | 113 | 3038 | 11110 | 27696 | 131 | 95500 | 210000 | 377250 |
| **27+0 to 30+6** | 277 | 3624 | 10630 | 26168 | 289 | 77400 | 192000 | 350200 |
| **31+0 to 34+0** | 761 | 6320 | 13370 | 25250 | 780 | 96475 | 216000 | 353525 |
|  | **Hemoglobin (g/dL)** | | | | **Hematocrit (%)** | | | |
| **Gestational age** | **n** | **2.5^th^** | **50^th^** | **97.5^th^** | **n** | **2.5^th^** | **50^th^** | **97.5^th^** |
| **23+0 to 26+6** | 131 | 11.4 | 15.5 | 20.8 | 131 | 32.2 | 44.4 | 57.2 |
| **27+0 to 30+6** | 289 | 12.6 | 17.5 | 22.1 | 289 | 36.3 | 48.5 | 61.2 |
| **31+0 to 34+0** | 780 | 13.3 | 18.2 | 23.3 | 780 | 36.9 | 50.0 | 63.6 |
|  | **Neutrophilic granulocytes (cells/µL)** | | | | **Lymphocytes (cells/µL)** | | | |
| **Gestational age** | **n** | **2.5^th^** | **50^th^** | **97.5^th^** | **n** | **2.5^th^** | **50^th^** | **97.5^th^** |
| **23+0 to 26+6** | 30 | 1135 | 6650 | 28643 | 30 | 286 | 2450 | 8508 |
| **27+0 to 30+6** | 66 | 1163 | 4850 | 24563 | 66 | 763 | 2750 | 7013 |
| **31+0 to 34+0** | 185 | 2560 | 7000 | 15540 | 182 | 1500 | 3400 | 6300 |

**Supplemental C_2.** 2.5th, 50^th^ and 97.5^th^ percentiles of hematological parameters (leukocytes, thrombocytes, hemoglobin, hematocrit, neutrophilic granulocytes, lymphocytes) for male preterm neonates, stratified in gestational age groups of 23+0 to 26+6 weeks, 27+0 to 30+6 weeks, and 31+0 to 34+0 weeks, on the first day after birth.

|  | **Leukocytes (cells/µL)** | | | | **Thrombocytes (cells/µL)** | | | |
| --- | --- | --- | --- | --- | --- | --- | --- | --- |
| **Gestational age** | **n** | **2.5^th^** | **50^th^** | **97.5^th^** | **n** | **2.5^th^** | **50^th^** | **97.5^th^** |
| **23+0 to 26+6** | 147 | 3023 | 10800 | 29005 | 162 | 66363 | 179000 | 331925 |
| **27+0 to 30+6** | 342 | 3546 | 9680 | 23165 | 358 | 86775 | 186000 | 336300 |
| **31+0 to 34+0** | 919 | 5798 | 12490 | 23165 | 928 | 93588 | 200000 | 33200 |
|  | **Hemoglobin (g/dL)** | | | | **Hematocrit (%)** | | | |
| **Gestational age** | **n** | **2.5^th^** | **50^th^** | **97.5^th^** | **n** | **2.5^th^** | **50^th^** | **97.5^th^** |
| **23+0 to 26+6** | 162 | 11.8 | 15.2 | 20.9 | 162 | 33.3 | 43.3 | 57.8 |
| **27+0 to 30+6** | 358 | 13.1 | 18.0 | 22.7 | 358 | 36.1 | 50.0 | 62.5 |
| **31+0 to 34+0** | 928 | 13.7 | 18.4 | 23.0 | 928 | 37.8 | 50.4 | 62.6 |
|  | **Neutrophilic granulocytes (cells/µL)** | | | | **Lymphocytes (cells/µL)** | | | |
| **Gestational age** | **n** | **2.5^th^** | **50^th^** | **97.5^th^** | **n** | **2.5^th^** | **50^th^** | **97.5^th^** |
| **23+0 to 26+6** | 44 | 1108 | 5000 | 23735 | 44 | 419 | 2650 | 5985 |
| **27+0 to 30+6** | 100 | 2219 | 4900 | 29120 | 100 | 1200 | 2650 | 5963 |
| **31+0 to 34+0** | 242 | 1908 | 6650 | 14893 | 238 | 1300 | 3150 | 5915 |

**Supplemental C_3_male versus female.** This table shows the Z-scores for the 2.5th, 50^th^ and 97.5^th^ percentiles of hematological parameters (leukocytes, thrombocytes, hemoglobin, hematocrit, neutrophilic granulocytes, lymphocytes), along with the critical values for males and females. The column Z-score indicates the Z-score for each sex, representing the deviation of the measurement from the mean of the reference population. The critical value column lists the threshold values set for each sex. The Z > Z_crit_ column indicates whether the Z-score for the respective blood parameter exceeds the critical value (False or True). The column s2/(s2−s1) <3 provides a calculation that checks specific statistical conditions. The "Male is normally distributed" and "Female is normally distributed p-value" columns show the p-values for the normal distribution of blood values for each sex, as determined by statistical tests.

|  | **Leukocytes (cells/µL)** | | | | | | **Thrombocytes (cells/µL)** | | | | | |
| --- | --- | --- | --- | --- | --- | --- | --- | --- | --- | --- | --- | --- |
| **Gestational age cohort**  **(male vs. female)** | **Z-score** | **critical value** | **Z>Z_crit​_** | **s2​/(s2​−s1​) <3** | **Male norm. distr. p-value** | **Female norm. distr. p-value** | **Z-score** | **critical value** | **Z>Z_crit​_** | **s2​/(s2​−s1​) <3** | **Male norm. distr. p-value** | **Female norm. distr. p-value** |
| **23+0 to 26+6** | 0.15 | 3.12 | False | True | 0.013 | 0.038 | 2.60 | 3.32 | False | True | 0.210 | 0.676 |
| **27+0 to 30+6** | 1.79 | 4.82 | False | True | 0.002 | 0.01 | 0.51 | 4.93 | False | True | 0.232 | 0.133 |
| **31+0 to 34+0** | 4.77 | 7.94 | False | True | 0.022 | 0.01 | 4.67 | 8.00 | False | True | 0.503 | 0.921 |
|  | **Hemoglobin (g/dL)** | | | | | | **Hematocrit (%)** | | | | | |
| **Gestational age cohort**  **(male vs. female)** | **Z-score** | **critical value** | **Z>Z_crit​_** | **s2​/(s2​−s1​) <3** | **Male norm. distr. p-value** | **Female norm. distr. p-value** | **Z-score** | **critical value** | **Z>Z_crit​_** | **s2​/(s2​−s1​) <3** | **Male norm. distr. p-value** | **Female norm. distr. p-value** |
| **23+0 to 26+6** | 0.26 | 3.32 | False | True | 0.604 | 0.894 | 0.46 | 3.32 | False | True | 0.446 | 0.986 |
| **27+0 to 30+6** | 2.00 | 4.93 | False | True | 0.562 | 0.701 | 1.32 | 4.92 | False | True | 0.690 | 0.984 |
| **31+0 to 34+0** | 2.08 | 8.00 | False | True | 0.498 | 0.598 | 0.88 | 800 | False | True | 0.955 | 0.752 |
|  | **Neutrophilic granulocytes (cells/µL)** | | | | | | **Lymphocytes (cells/µL)** | | | | | |
| **Gestational age cohort**  **(male vs. female)** | **Z-score** | **critical value** | **Z>Z_crit​_** | **s2​/(s2​−s1​) <3** | **Male norm. distr. p-value** | **Female norm. distr. p-value** | **Z-score** | **critical value** | **Z>Z_crit​_** | **s2​/(s2​−s1​) <3** | **Male norm. distr. p-value** | **Female norm. distr. p-value** |
| **23+0 to 26+6** | 0.86 | 1.67 | False | True | 0.02 | 0.146 | 0.36 | 1.67 | False | True | 0.092 | 0.071 |
| **27+0 to 30+6** | 0.88 | 2.50 | False | True | <0.001 | 0.007 | 0.69 | 2.50 | False | True | 0.065 | 0,122 |
| **31+0 to 34+0** | 1.43 | 4.00 | False | True | 0.137 | 0.044 | 1.77 | 3.97 | False | True | 0.07 | 0.377 |
